# Dietary Restrictions and Depressive Symptoms: Longitudinal Results from the Constances Cohort

**DOI:** 10.3390/nu12092700

**Published:** 2020-09-04

**Authors:** Joane Matta, Nicolas Hoertel, Guillaume Airagnes, Sebastien Czernichow, Emmanuelle Kesse-Guyot, Frederic Limosin, Marcel Goldberg, Marie Zins, Cédric Lemogne

**Affiliations:** 1 Inserm, Cohortes Epidémiologiques en Population, UMS 011, 94800 Villejuif, France; guillaume.airagnes@aphp.fr (G.A.); marcel.goldberg@inserm.fr (M.G.); marie.zins@inserm.fr (M.Z.); 2 Faculté de Santé, Université de Paris, UFR de Médecine, 75006 Paris, France; nicolas.hoertel@aphp.fr (N.H.); sebastien.czernichow@aphp.fr (S.C.); frederic.limosin@aphp.fr (F.L.); cedric.lemogne@aphp.fr (C.L.); 3 Service de Psychiatrie et d’Addictologie de l’adulte et du sujet âgé, Hôpital Corentin-Celton, AP-HP.Centre—Université de Paris, 75015 Paris, France; 4 Institut de Psychiatrie et Neurosciences de Paris (IPNP), Université de Paris, INSERM, UMR_S1266, 75014 Paris, France; 5 Centre Ambulatoire d’Addictologie, Hôpital européen Georges-Pompidou, AP-HP.Centre—Université de Paris, 75015 Paris, France; 6 Dép artement de Nutrition, Centre Spécialisé Obésité IdF, Hôpital européen Georges-Pompidou, AP-HP.Centre—Université de Paris, 75015 Paris, France; 7 Sorbonne Paris Nord Université, Inserm, Inrae, Cnam, Nutritional Epidemiology Research Team (EREN), Epidemiology and Statistics Research Center—Université de Paris (CRESS), 93017 Bobigny, France; e.kesse@eren.smbh.univ-paris13.fr; 8 Service de Psychiatrie de l’adulte, Hôpital Hôtel-Dieu, AP-HP.Centre—Université de Paris, 75004 Paris, France

**Keywords:** depressive symptoms, dietary exclusions, prospective, epidemiology, nutrition

## Abstract

Cross-sectional results have suggested a linear association between the number of dietary exclusions and depressive symptoms. This longitudinal study aimed to examine the direction of this association. **Methods**: In the population-based Constances cohort, depressive symptoms were defined by a score ≥19 on the Centre of Epidemiologic Studies-Depression (CES-D) scale. Diet was measured with a 24-item qualitative food frequency questionnaire (FFQ). Both variables were available at inclusion (from 2012 to 2014) and on follow-up (2015 for the CES-D and 2017 for diet). Food exclusion was categorized into five different groups: No exclusion, exclusion of one, two, three, or ≥4 food groups according to the self-reported number of food groups rarely or never consumed. Logistic regressions were conducted, either taking depressive symptoms as the outcome on follow-up with dietary exclusions at baseline as predictor or with the opposite, adjusting for age, sex, education, income, alcohol intake, smoking, physical activity, and anemia. The path analysis included outcomes and covariates in one model. **Results**: The median follow-up was three years. A total of 29,337 participants (53.4% women, 48.15 ± 12.9 y.o.) had complete CES-D data and 25,356 (53.56% women, 49.05 ± 12.8 y.o.) FFQ data. Dietary exclusion at inclusion predicted depressive symptoms at follow-up (odds ratio [95% confidence interval]: 2.35 [1.62–3.40] for ≥4 excluded items compared to no exclusions). Depressive symptoms at inclusion predicted dietary exclusions at follow-up (3.45 [1.93–6.16] for ≥4 excluded items). In the path analysis, the standardized estimate of the association between dietary exclusions at inclusion and depressive symptoms at follow-up was by far higher than the opposite (0.1863 and 0.00189, respectively, both *p* < 0.05). **Conclusions**: The association of dietary exclusion with subsequent depression is stronger than the opposite association.

## 1. Introduction

Many epidemiological investigations have studied the association of diet with depressive symptoms but the results have been inconsistent; with some showing a protective effect of some dietary patterns such as the Mediterranean diet [1] or the healthy/prudent diet [2,3] or some food groups [4] such as high intakes of fruit, vegetables, fish, and whole grains that may be associated with a reduced depression risk and others showing no association [5,6]. The no association with depression might occur because of the focus on different types of dietary patterns such as the ‘varied diet’; moreover, the associations with depression were non-significant after controlling for age and sex in the latter studies.

A meta-analysis regarding diet and mental health suggested that a healthy pattern may decrease the risk of depression, whereas a western-style may increase the risk of depression. A dietary pattern characterized by a high intake of fruit, vegetables, whole grain, fish, olive oil, low-fat dairy and antioxidants, and low intakes of animal foods was associated with a decreased risk of depression. A dietary pattern characterized by a high consumption of red and/or processed meat, refined grains, sweets, high-fat dairy products, butter, potatoes and high-fat gravy, and low intakes of fruits and vegetables was associated with an increased risk of depression [7]. A limitation in most of the studies is that they have focused on the consumption of certain dietary patterns or food groups and their association with depressive symptoms but did not consider the impact of food removal from the diet; moreover, dietary exclusion trends such as vegetarianism have become frequently adopted nowadays which merits attention on its association with depression. We have recently shown using cross-sectional data from the French Constances cohort, a positive linear association between vegetarianism, i.e., a form of dietary exclusion with depressive symptoms [8]. In that study, depressive symptoms were associated with the exclusion of any food group and the estimates increased with the number of excluded food items, regardless of the type of excluded food items. These associations remained significant after adjustment for several potential confounders such as age, income, education, alcohol intake, smoking, physical activity, and chronic conditions such as anemia [8].

Although much emphasis has been placed on the potential impact of diet on depressive symptoms, the association between diet and depression may be bidirectional [9,10,11]. For instance, a retrospective study showed that vegetarians displayed elevated prevalence rates for depressive disorders and that the onset of these disorders tended to precede the onset of vegetarian diet [9]. Some other studies found evidence for a bidirectional association between depressive symptoms and dietary changes. For instance, a longitudinal study on fish consumption and depression in Australian adults have shown that for women, each additional weekly serving of fish consumed at baseline decreased the risk of having a new depressive episode by 6% (adjusted relative risk = 0.94, 95% confidence interval: 0.87, 1.01). However, fish consumption was not associated with depression in men. Another cross-sectional study on diet quality and mental health conducted on Canadian adults aged 18 and older has shown that fruit and vegetable consumption was inversely associated with depression and psychological distress; the association was attenuated after controlling for health-related factors [10,11].

For instance, data from the Invecchiare in Chianti study showed that fish and sweet food intakes were associated with a three-year improvement and deterioration in depressive symptoms, respectively, whereas depressive symptoms were associated with three-year changes in vegetable, meat, dairy product, and savory snack intakes [12]. However, to our knowledge, no study has yet examined bidirectional associations between depressive symptoms and food exclusions per se; i.e., irrespective of the type of food being excluded. Indeed, even though diet may have an impact on depressive symptoms, depression could also affect eating behavior. First, it is noteworthy that abnormal eating behavior belongs to the core diagnostic criteria of major depression. Both decreased and increased appetite may be observed, with a significant loss or gain of weight [13]. Alterations within the brain reward system may underlie the two opposite patterns, as increased appetite can be viewed as a compensatory behavior in response to the decreased rewarding value of food. However, it is unclear whether such changes in appetite translate into food items exclusion, that is whether changes in appetite lead to removal of some foods from the diet. Second, it is also a possibility that some people with depression try to adopt dietary changes as a way of treating their mental illness [13], they might have heard about some benefits of vegetarian diets on physical health and expect similar benefits on mental health [13].

Finally, this association could be explained to some extent by individuals presenting with eating disorders. A meta-analysis showed that eating disorders were risk factors for depression and that depression was a risk factor for eating disorders, especially in younger individuals [14]. The association between eating disorders and depression is complex and defined by intertwined physiological and behavioral factors that affect aetiology, symptomatology, response to treatment, and prognosis [15].

Some covariates such as anemia and physical activity might play a role in the association between diet and depressive symptoms. In fact, anemia is associated with both diet and depression [16,17,18]. In a study on French individuals, depressed participants were significantly more likely to have anemia compared to non-depressed participants, even after adjustment for sociodemographic and health-related variables. Anemia prevalence increased with depression severity, suggesting a dose-response relationship. Moreover, iron and vitamin C consumption were negatively associated with haemoglobin levels, on the other side; a westernized diet was associated with higher risk of anemia.

Given the scarcity of results in this research area, our main objective was to assess the main direction of the association between depressive symptoms and dietary exclusions, while adjusting for potential confounders including sociodemographic characteristics and lifestyle behaviors. We hypothesized that both directions would be observed and we aimed at estimating their relative effect sizes. Answering this question would critically inform prevention strategies including the information campaign.

To this aim, since there were two outcomes at follow-up, two separate logistic regression models were conducted as a first step: One having depressive symptoms as the dependent variable and the other dietary exclusions as the dependent variable. We then used the path analysis in order to test the bidirectional associations between depressive symptoms and dietary exclusions, and quantify their respective magnitude. The path model aimed at determining the direction of the association by quantifying the two paths simultaneously while taking into consideration all variables with baseline and follow-up values in one model.

## 2. Methods

### 2.1. Population

Constances is a large, population-based, prospective cohort whose recruitment began in 2012 and ended in 2019 with a total size of more than 200,000 subjects, including volunteers aged 18 to 69 years at baseline and living in 21 selected departments (administrative divisions) throughout metropolitan France, in both rural and urban settings [19]. The Constances cohort study has received the authorization of the French Data Protection Authority (Commission Nationale de l’Informatique et des Libertés, CNIL) and the institutional review board of the National Institute for Medical Research (Inserm) (Authorization number 910486). All subjects included in this study gave their informed consent.

An annual self-administered follow-up questionnaire was then completed by participants at home, using either a paper questionnaire or internet. Depressive symptoms were assessed at both inclusion and in the 2015 annual questionnaire. The dietary intake was assessed at inclusion and in the 2017 annual questionnaire.

Inclusion criteria included having had a follow-up in 2015 for depressive symptoms and in 2017 for dietary intake and a baseline data collection in the years of 2012–2014. There were no exclusion criteria. Supplementary Figure S1 displays the flowchart for the selection of participants.

### 2.2. Variables

Both depressive symptoms and dietary exclusions were treated as predictors and outcomes in separate logistic regression models.

#### 2.2.1. Depressive Symptoms at Baseline and during the Follow-Up

Depressive symptoms were measured with the self-administered Center of Epidemiologic Studies Depression scale (CES-D). Depressive symptoms were modeled as a binary variable defined by a CES-D score ≥19, following the validated cutoff of the French version (sensitivity/specificity for the diagnosis of major depression: 0.85/0.86) [20] and as continuous in the path analysis model.

#### 2.2.2. Dietary Exclusions Measured at Baseline and during the Follow-Up

The diet type information was obtained using a 24-item qualitative food frequency questionnaire that was developed according to the French National Nutrition and Healthy Program and was developed in Constances in a way to make it comparable to other national data. It has already been used in the same context in Constances [21,22]. The consumption of each food item on the questionnaire was measured using the question: “Usually, how frequently do you consume this item (irrespective of the food preservation method, the time and the place of consumption)?” on a six-point scale ranging from never to more than once per day. No specific timeframe was mentioned.

Questions referring to exclusions of food groups namely fish, eggs, meat, poultry, milk and dairies, fruits, vegetables and legumes intake were available and grouped into eight food groups: (1) Fish: All fish and seafood products; (2) Meat: All meat and poultry products including beef, lamb, pork, and cold cuts; (3) Eggs; (4) Fruits: All types raw and cooked including fruit juices; (5) Vegetables: All types raw and cooked including vegetable juices; (6) Milk and dairies: Milk skimmed, reduced fat or full-fat, and all types of cheese; (7) Legumes: Lentils, chickpeas, and beans; (8) Bread and cereals: White or whole wheat bread, toast, cereals, and pasta.

For each group, individuals answering never or almost never were considered as not consuming the food items which were coded as binary variables (never/almost never *versus* consumed regardless of frequency of use). The total number of exclusions was calculated as the sum of all dietary exclusions and was further categorized into five different groups: No exclusion, one, two, or three exclusions, and four exclusions or more based on the distribution of this variable.

Dietary exclusions were modeled as a categorical variable in all models.

The food groups and food items that were chosen are based on the regular food consumption of French individuals and cover the intake of the three energy yielding macronutrients: Carbohydrates, proteins, and fatty acids. They constitute the basis of a regular diet and are part of the recommended intake in the French National Nutrition and Health Program. There was no specific focus on the foods that were chosen but rather a purpose of including all possible items that constitute a regular dietary intake, in order to test the effect of their exclusion on depressive symptoms.

#### 2.2.3. Covariates Measured at Baseline

##### Anemia

Individuals were considered as having anemia at baseline if they had a hemoglobin <130 g/L for men and <120 g/L for women.

##### Physical Activity

Physical activity is associated with depression [23].

Physical activity outside work was analyzed as a score ranging from zero to six, zero being inactive and six being very active. It was calculated using three main questions from the Constances questionnaire in two steps.

First, an intermediate score is calculated for each of the following questions:

(1) In the past 12 months have you regularly engaged in gardening, cleaning, or handy work? (2) In the past 12 months have you regularly practiced a sport (aside from gardening, cleaning, or handy work)? (3) In the past 12 months have you regularly gone biking or on walking trips (for work or leisure)? The attributed intermediate score is: Zero if the answer is no, one if the answer is: “Yes, less than 15 min” or “yes, less than 2 h” depending on the question, two if the answer is “Yes, 15 min and more” or “yes, 2 h and more” depending on the question.

Second, a summed score of the three intermediate scores is calculated when the three questions have been answered. If one answer is missing, the data for physical activity is considered as missing.

This score has been used for the assessment of physical activity in relation with coronary events in France using data from the MONICA study [24].

##### Other Covariates

Education is associated with depression [25]. Education was categorized into four levels: Less than or equal to high school diploma, undergraduate degree, and postgraduate degree or other. Household monthly income was categorized into three levels: <1500, 1500–2800, and ≥2800 euros. Smoking was categorized into never, current, or former smokers. Alcohol intake was categorized as one time per week or more; 2–3 times per month, one time per month or less and never. Age and sex were also obtained at baseline.

The variables: “Are you eating to stay healthy” and “Currently are you on a diet” were measured as ‘yes’ or ‘no’. 

##### Missing Data

The main purpose of this paper is to determine the direction of the association between diet and depressive symptoms. We have conducted two separate series of logistic regression models with depressive symptoms being the outcome at first and dietary exclusions being the outcome at second. Since depressive symptoms and dietary exclusions were not measured at the same year of follow-up; complete data were slightly different in the two logistic regression models as follows:

From 35,001 participants included in 2012–2014 and with data on depressive symptoms at follow-up in 2015; 83.8% (N = 29,337) were included in the present analysis because they had no missing data in any of the variables in the models considering dietary exclusions at baseline as the predictor and depressive symptoms at follow-up as the outcome.

From 29,141 participants included in 2012–2014 and with data on dietary exclusions at follow-up; 80.1% (N = 25,356) were included in the present analysis because they had no missing data in any of the variables in the models considering depressive symptoms at baseline as the predictor and dietary exclusions at follow-up as the outcome.

Further logistic regression analyses considering individuals with missing values and not restricted to individuals with complete data in models 1 and 2 were computed.

### 2.3. Statistical Analyses

Descriptive statistics were performed to provide characteristics of the sample by depressive symptoms at follow-up and dietary exclusions at follow-up, and are presented as percentages or means ± standard deviations (SD) as appropriate.

As already mentioned, since there were two outcomes at follow-up, two separate logistic regression models were conducted prior to the path analysis: One having depressive symptoms as the dependent variable and the other dietary exclusions as the dependent variable.

All statistical analyses were carried out with the Statistical Analysis System (SAS, version 9.4, Cary, NC, USA). All logistic regression analyses were carried using the listwise exclusion treatment with SAS proc logistic.

The path analysis for the path model was conducted with SAS Proc Calis. The statistical significance was evaluated with the α set a priori at 0.05.

#### 2.3.1. Depressive Symptoms at Follow-Up

The association between dietary exclusions at baseline and depressive symptoms at follow-up was estimated with odds ratios (OR) and their 95% confidence intervals (CI) were computed through logistic regression models. The first model was adjusted for age and sex, the second for model 1 adding all covariates (physical activity level, smoking status, alcohol intake, anemia, income, and education).

#### 2.3.2. Dietary Exclusions at Follow-Up

The association between depressive symptoms at baseline and dietary exclusions at follow-up was estimated with odds ratios (OR) and their 95% confidence intervals (CI) were computed through multinomial logistic regression analysis models. The first model was controlled for age and sex, the second for model 1 adding all covariates (physical activity level, smoking status, alcohol intake, anemia, income, and education).

#### 2.3.3. Path Analysis

Mediation analyses for the path effects between diet and depressive symptoms were conducted in order to assess the strength of the direct association between the two main predictors/outcomes of interest. In the mediation model, depressive symptoms and dietary exclusions were analyzed as continuous variables. Dietary exclusions and depressive symptoms at baseline and follow-up were included as well as all covariates; covariates were allowed to correlate.

The path analysis cross-lagged model would take all our variables (at baseline and at follow-up) together, along with the covariates, in one model. It allows the investigation of the reciprocal causal effects in the model. Standardized estimates can be compared and can be used to determine causal predominance. This model is very frequently used when the datasets consist of only two waves of data. The effects of the path analysis presented here are direct and indirect effects that take into consideration within and between-subject as well as bi-directional effects.

In the path model, because we sought to examine simultaneously all path coefficients, no paths in any of the models were fixed to zero. The model fitted well: CFI = 0.99, TLI = 0.99, RMSEA = 0.01 [26,27].

#### 2.3.4. Supplementary Analyses

We ran two kinds of supplementary analyses. First, we were interested in testing whether our results were specific to depressive symptoms. Second, we conducted a series of sensitivity analyses to assess the robustness of our results.

##### Specificity Analyses

In order to examine if the association between dietary exclusions and depressive symptoms are specific to the former variable or could also be found using other health conditions; we have examined the role of self-rated health in the path analysis model instead of depressive symptoms. Self-rated health was thus entered into the model at follow-up in the same year of depressive symptoms (i.e., in 2015). Assessment of self-rated health is performed by use of a standard measure included in the yearly questionnaires sent to all participants: “How would you judge the state of your general health?”. The participants respond on an eight-point Likert scale (1 = very good, 8 = very poor). Self-rated health was analyzed as a continuous variable.

##### Sensitivity Analyses

Further logistic regression analyses not restricted to individuals with complete data in both models 1 and 2 were computed. To test the robustness of our results, we reproduced the analyses that comprised depressive symptoms as a binary variable while analyzing it as a continuous variable. General linear models were computed with depressive symptoms at follow up being the dependent variable and logistic regression adjusting for continuous depressive symptoms at baseline when dietary exclusions at follow-up were the outcome. Continuous depressive symptoms were divided by their interquartile range in the analyses of the multinomial logistic regression so that our analysis offers the possibility to compare an individual in the middle of the upper half of the predictor’s distribution with an individual in the middle of the lower half.

Interactions with time were added to the logistic regressions model (i.e., when considering either depressive symptoms or dietary exclusions as dependent variables).

Interactions with the variables “Currently, are you on a diet?” and “Eating to stay healthy” were tested separately by adding this variable and the related interaction term to the logistic regression models (i.e., when considering either depressive symptoms or dietary exclusions as dependent variables).

Finally, a path model considering associations between depressive symptoms and dietary exclusions at follow-up was conducted. The path model includes both depressive symptoms and dietary exclusions at both times.

## 3. Results

Table 1 presents the characteristics of the 29,337 study participants with complete data for depressive symptoms at follow-up as the outcome. Participants with depressive symptoms were more frequently women, smokers, and had more frequently a low income, a low education, a low physical activity, and had more dietary exclusions at baseline.

Table 2 presents the characteristics of 25,356 by dietary exclusions at follow-up. Participants with dietary exclusions had more frequently depressive symptoms, had more frequently lower income, low education, higher rates of smoking, and lower physical activity levels.

The results of the logistic regressions that were done prior to the path analysis are presented in Table 3 and Table 4.

Table 3 shows OR and 95% CI for the probability of having depressive symptoms at follow-up associated to dietary exclusions at baseline. In models 1 and 2, individuals had a higher odd of depressive symptoms at follow-up as soon as one dietary exclusion was adopted (model 1: OR [95% CI]: 3.47 [2.41–4.99] and model 2: OR = 2.35 (1.62–3.40) for ≥ 4 exclusions compared to no exclusion). In addition, the odds increased with the number of exclusions with significant linear trends (*p* for trend < 0.0001).

Table 4 shows OR and 95% CI from multinomial regression of having dietary exclusions at follow-up associated to depressive symptoms at baseline (N = 25,356). In models 1 and 2, participants with depressive symptoms at baseline had higher odds of having dietary exclusions at follow-up (model 1: OR [95% CI]: 5.56 [3.18–9.74] for ≥4 exclusions; model 2: OR [95% CI]:3.45 [1.93–6.16] for ≥4 exclusions compared to no exclusion).

**Figure 1:**
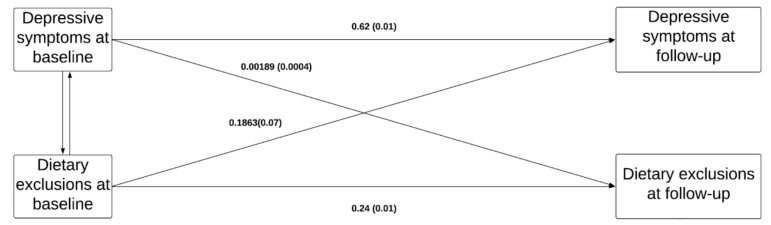
Depressive states defined by a Centre of Epidemiologic Studies-Depression (CES-D) score and analyzed as a continuous variable. Dietary exclusions defined as the number of exclusions and analyzed as a continuous variable. Fit statistics: CFI = 0.99, TLI = 0.99, RMSEA = 0.01.

Results of the path model (N = 22,815) are presented in Figure 1. While both paths were significant, the direct effect between dietary exclusions at baseline and depressive symptoms at follow-up was about 100 times greater than the direct effect from depressive symptoms at baseline to dietary exclusions at follow-up [estimate (SD) 0.1863 (0.07) *versus* 0.00189 (0.0004) *p* < 0.0001].

### Supplementary Analyses

Supplementary Table S1 shows OR and 95% CI of having depressive symptoms at follow-up, the main predictor being dietary exclusions but not restricted to complete data in models 1 and 2. This did not change the interpretation of the results.

Supplementary Table S2a shows estimates and their standard errors from general linear models when using the CES-D score at follow-up as a continuous variable. This did not change the interpretation of the results.

Supplementary Table S2b displays results of the same analyses as Supplementary Table S2a but is not restricted to complete data in models 1 and 2. There was no difference in the overall significance. Supplementary Table S3 shows OR and 95% CI from multinomial logistic regression of having dietary exclusions at follow-up associated to depressive symptoms at baseline not restricted to complete data in models 1 and 2 which do not change the results either.

Analyses shown in Table 4 and in Supplementary Table S3 were repeated using CES-D at baseline as a continuous variable and are shown in Supplementary Table S4a,b. Significant results remained unchanged.

Models including number of years of follow-up were also tested, but interactions with duration in the logistic regression models were not significant so we did not proceed into including further interactions of time in the path analysis model.

Additionally, models including the variables “Are you on a diet?” or “Eating to stay healthy” were computed, but interactions with these variables in the logistic regression models were not significant so we did not proceed into including further interactions in the path analysis model.

Results from the path analysis model considering self-rated health instead of depressive symptoms were not significant (i.e., there was no significant effect from self-rated health at baseline on dietary exclusions at follow-up neither an effect from dietary exclusions at baseline to self-rated health at follow-up), suggesting that our significant results obtained with depression were not non-specific.

## 4. Discussion

### 4.1. Main Findings

In a large population-based cohort, as soon as there was a dietary exclusion, the odds of having depressive symptoms were significant and increased linearly with the number of exclusions. The odds of dietary exclusions at follow-up were also associated with depressive symptoms at baseline and increased linearly with increasing depressive symptoms, supporting a bidirectional relationship between depressive symptoms and dietary exclusions.

However, the path analysis model showed that although both these paths were significant, the magnitude of effect of dietary exclusions on depressive symptoms was much greater than that of depressive symptoms on dietary exclusions, suggesting that the latter might precede the occurrence of depressive symptoms and that the effect of depressive symptoms on dietary exclusions may be less clinically significant. Sensitivity path analyses yielded similar results, supporting the robustness of our findings.

### 4.2. Strengths and Limitations

The strengths of our study include the large sample and the design of the Constances cohort that allowed us to study the path model with both predictors and outcomes at two time points. To our knowledge, this is the first study to quantify the direction of the association of dietary exclusions and depressive symptoms in a large population-based sample. This study also assessed a wide range of eating behavior allowing a broader picture of dietary exclusions and mental health. Limitations include the observational nature of the data, which restricts the possibility of drawing causal conclusions with regard to associations between dietary exclusions and depressive symptoms. However, both outcomes/predictors of interest (i.e., depressive states and dietary exclusions) were measured at both baseline and follow-up so we had a prospective understanding of the variables included in the model, but only two time points were available, which limited the use of different statistical approaches.

Second, the FFQ does not provide information on dietary quantities so the lack of measure of calories prevents the dissociation of food exclusion from food restriction. We acknowledge that the lack of the amount of nutrients and total caloric intake limits our understanding of the association between diet and depressive symptoms on the long term, however this was not possible given the qualitative nature of dietary intake in the Constances questionnaire.

Third, major depression was not assessed in the present study. Although our main proxy for depression was based on a well-validated measure of depressive states (20), future studies are needed to examine whether our results are also observed in clinical populations of individuals with major depression.

Fourth, eating disorders were not assessed in the study. However, should eating disorders explain the association between dietary exclusions and depressive symptoms, one may assume that eating disorders occur before dietary exclusions and then lead to depressive symptoms, which is in line with our finding that the main direction of the bidirectional association is greater from dietary exclusions to depressive symptoms than the opposite direction.

### 4.3. Explanatory Hypotheses

Several factors may underlie the direction from dietary exclusions to depressive symptoms. Anemia, which might be initiated by dietary exclusions and may result from folate, B6, iron, or B12 deficiencies, is associated to depressive symptoms [16]. However, the present analyses do not support this explanation since the analyses were adjusted for anemia. Furthermore, we have conducted supplementary analyses and removed individuals with anemia from the analyses, but this did not change the direction and the interpretation of the results (data not shown). Therefore, it is unlikely that the deficiencies in folate, iron, vitamins B6 and B12 explain the association between dietary exclusions and depressive symptoms through anemia.

The association between dietary exclusions and depressive symptoms could be explained by nutrient deficiencies that are not related to anemia such as omega-3 [28], zinc, magnesium, selenium, calcium, and vitamin D, etc. [29,30,31]. Results from the SuViMax cohort have suggested that vitamin D can have a protective effect with regard to depressive symptoms especially among individuals with a low overall diet quality [32]. Vitamin D plays multiple roles in the organism, particularly the central nervous system and depressive symptoms. The latter association can further be modulated by diet.

Folate deficiency had been associated with decreased effectiveness of antidepressants and thus cognitive decline, moreover, folate supplementation has provided a potential benefit on mood during the year after the first depressive episode. Meta-analysis on omega-3 fatty acids, mainly EPA and DHA has shown a positive effect on mood, possibly through neuronal membrane stability, signal transduction, and serotonin uptake among others. However, the randomized double blind placebo trial SU.FOL.OM3 has shown that, after a median of 4.7 years of supplementation of folate and B-vitamins and/or omega-3 fatty acids, there was no association between vitamins B and depression, with results being positive (i.e., negative association with depressive symptoms) in terms of omega-3 fatty acids on depressive symptoms in men but not in women [33].

Indeed, with the number of food items exclusion in the present study grows the risk of detrimental interactions of multiple deficiencies. In 2014, a meta-analysis has shown that consumption of fruits, vegetables, and whole grains was associated to a reduced risk of depression; additionally, the consumption of a Mediterranean diet type has been lately suggested as a potential factor in reducing depression in meta-analyses using data from longitudinal studies [3]. These exclusions may create deficiencies of beneficial nutrients that may play a role in enhancing the mood observed while adopting healthy diets such as the Mediterranean one. It was not possible for us to test the association of nutrient deficiencies with depressive symptoms as this was not available in the Constances data. However, since our results show that dietary exclusions precede the development of depressive symptoms, they suggest that clinicians must assess the eating behavior of their patients while counseling and check for potential food groups exclusions.

Finally, the association between dietary exclusions and depressive symptoms could be explained to some extent by individuals presenting with eating disorders. A meta-analysis showed that eating disorders were risk factors for depression and that depression was a risk factor for eating disorders, especially in younger individuals [14].

In terms of public health interest, depression is the leading cause of disability worldwide. Our results suggest the potential value to focus on protective dietary patterns in order to help prevent the occurrence of depression. Importantly, our findings suggest that this focus should be done on the prevention of food items exclusions rather than specific food types to prevent it. Clinicians can be advised to detect food exclusion patterns among their patients in order to possibly minimize its association in the development of depressive symptoms.

In conclusion, dietary exclusions may be a substantial risk factor for depression. Other longitudinal studies and randomized controlled trials of interventions on dietary patterns are needed to support our findings.

## Figures and Tables

**Table 1:** Participant’s baseline characteristics by depressive symptoms at follow-up (N = 29,337).

	All (%)	No Depressive Symptoms at Follow-Up (CES-D Score <19)	Depressive Symptoms at Follow-Up (CES-D Score ≥19)	*p*-Value (Depressive Symptoms *versus* No Depressive Symptoms)
**Women, N (%)**	15,671 (53.42)	12,223 (50.57)	3448 (66.74)	<0.0001
**Age, mean (SD)**	49.14 (12.91)	47.88 (12.49)	49.41 (12.98)	<0.0001
**Household income, N (%)**				<0.0001
<1500 euros or does not want to answer	3562 (12.14)	2544 (10.53)	1018 (19.71)	
1500 to less than 2800 euros	7419 (25.29)	5793 (23.97)	1626 (31.48)	
≥2800 euros	18,356 (62.57)	15,834 (65.51)	2522 (48.82)	
**Education level, N (%)**				<0.0001
≤ high school diploma or other diploma	11,515 (39.25)	9225 (38.17)	2290 (44.33)	
Undergraduate degree	10,622 (36.21)	8749 (36.20)	1873 (36.26)	
Postgraduate degree	7200 (24.54)	6197 (26.64)	1003 (19.42)	
**Depressive symptoms (CES-D score ≥19), N (%)**	4066 (13.86)	1614 (6.68)	2452 (47.46)	<0.0001
**Dietary exclusions, N (%)**				<0.0001
No exclusion	21,844 (74.16)	18,305 (75.73)	3539 (68.51)	
1 exclusion	5716 (19.48)	4538 (18.77)	1178(22.80)	
2 exclusions	1342 (4.57)	1039 (4.30)	303 (5.87)	
3 exclusions	309 (1.05)	214 (0.89)	95 (1.84)	
≥4 exclusions	126 (0.43)	75 (0.31)	51 (0.99)	
**Alcohol intake, N (%)**				<0.0001
1/week or more	18,372 (62.62)	15,453 (63.93)	2919 (56.50)	
2–3/months	5987 (20.41)	4916 (20.34)	1071 (20.73)	
1/month or less	3857 (13.15)	2980 (12.33)	877 (16.98)	
Never	1121 (3.82)	822 (3.40)	299 (5.79)	
**Smoking status, N (%)**				<0.0001
Non-Smokers	13,561 (46.22)	11,365 (47.02)	2196 (42.51)	
Smokers	4915 (16.75)	3721 (15.39)	1194 (23.11)	
Ex-smokers	10,861 (37.02)	9085 (37.59)	1776 (34.38)	
**Physical activity level, N (%)**				<0.0001
Low	7615 (25.95)	5918 (24.48)	1697 (32.85)	
Moderate	13,569 (46.25)	11,260 (46.58)	2309 (44.70)	
High	8153 (27.79)	6993 (28.93)	1160 (22.45)	
**Anemia, N (%)**	818 (2.78)	644 (2.66)	174 (3.37)	0.005

CES-D—Center of Epidemiologic Studies Depression scale (CES-D).

**Table 2:** Participant’s baseline characteristics by dietary exclusions at follow-up (N = 25,356).

	All (%)	No Exclusion	1 Exclusion	2 Exclusions	3 Exclusions	≥4 Exclusions	*p*-Value
**Women, N (%)**	13,580 (53.5)	11,957 (53.32)	1284 (56.44)	261 (55.18)	62 (48.06)	16 (30.19)	0.0002
**Age, mean (SD)**	49.9 (12.8)	50.60 (12.65)	45.73 (12.90)	43.46 (13.07)	45.25 (13.06)	41.75 (12.12)	<0.0001
**Household income, N (%)**							<0.0001
<1500 euros or does not want to answer	2902 (11.4)	2365 (10.55)	375 (16.48)	107 (22.62)	38 (29.46)	17 (32.08)	
1500 to less than 2800 euros	6354 (25.0)	5407 (24.11)	724 (31.82)	161 (34.04)	40 (31.01)	22 (41.51)	
≥2800 euros	16,100 (63.4)	14,654 (65.34)	1176 (51.69)	205 (43.34)	51 (39.53)	14 (26.42)	
**Education level, N (%)**							<0.0001
≤ high school diploma or other diploma	10,005 (39.4)	8699 (38.79)	992 (43.60)	213 (45.03)	66 (51.16)	35 (66.04)	
Undergraduate degree	9069 (35.7)	8023 (35.78)	824 (36.22)	170 (35.94)	36 (27.91)	16 (30.19)	
Postgraduate degree	6282 (24.7)	5704 (25.43)	459 (20.18)	90 (19.03)	27 (20.93)	2 (3.77)	
**Depressive symptoms (CES-D score ≥19), N (%)**	3350 (12.2)	2771 (12.36)	431 (18.95)	96 (20.30)	31 (24.03)	21 (39.62)	<0.0001
**Dietary exclusions at baseline, N (%)**							<0.0001
No exclusion	19,069 (75.2)	17,852 (79.60)	1012 (44.48)	167 (35.31)	30 (23.26)	8 (15.09)	
1 exclusion	4829 (19.0)	3787 (16.89)	856 (37.63)	137 (28.96)	40 (31.01)	9 (16.98)	
2 exclusions	1126 (4.4)	657 (2.93)	309 (13.58)	117 (24.74)	29 (22.48)	14 (26.42)	
3 exclusions	251 (0.98)	106 (0.47)	84 (3.69)	33 (6.98)	15 (11.63)	13 (24.53)	
≥4 exclusions	81 (0.31)	24 (0.11)	14 (0.62)	19 (4.02)	15 (11.63)	9 (16.98)	
**Alcohol intake, N (%)**							<0.0001
1/week or more	15,911 (62.7)	14,330 (63.90)	1245 (54.73)	243 (51.37)	66 (51.16)	27 (50.94)	
2–3/months	5214 (20.5)	4573 (20.39)	495 (21.76)	105 (22.20)	29 (22.48)	12 (22.64)	
1/month or less	3302 (13.0)	2785 (12.42)	400 (17.58)	83 (17.55)	23 (17.83)	11 (20.75)	
Never	929 (3.6)	738 (3.29)	135 (5.93)	42 (8.88)	11 (8.53)	3 (5.66)	
**Smoking status, N (%)**							<0.0001
Non-Smokers	11,937 (47.0)	10,671 (47.58)	976 (42.90)	206 (43.55)	63 (48.84)	21 (39.62)	
Smokers	3916 (15.4)	3264 (14.55)	493 (21.67)	106 (22.41)	31 (24.03)	22 (41.51)	
Ex-smokers	9503 (37.4)	8491 (37.86)	806 (35.43)	161 (34.04)	35 (27.13)	10 (18.87)	
**Physical activity level, N (%)**							<0.0001
Low	6368 (25.1)	5391 (24.04)	737 (32.40)	152 (32.14)	56 (43.41)	32 (60.38)	
Moderate	11,721 (46.2)	10,403 (46.39)	1059 (46.55)	199 (42.07)	45 (34.88)	15 (28.30)	
High	7267 (28.6)	6632 (22.57)	479 (21.05)	122 (25.79)	28 (21.71)	6 (11.32)	
**Anemia, N (%)**	694 (2.73)	580 (2.59)	86 (3.78)	21 (4.44)	5 (3.88)	2 (3.77)	0.0018

**Table 3:** Odds ratio (OR) and 95% confidence intervals (CI) from logistic regression for the odds of having depressive symptoms (binary measure) at follow-up associated to dietary exclusions at baseline (N = 29,337).

Exclusions at Baseline	Model 1	Model 2
No dietary exclusions	Ref	Ref
One exclusion	1.30 (1.19–1.38)	1.18 (1.09–1.27)
Two exclusions	1.47 (1.28–1.68)	1.23 (1.07–1.42)
Three exclusions	2.30 (1.79–2.95)	1.64 (1.27–2.11)
Four and more	3.47 (2.41–4.99)	2.35 (1.62–3.40)

Dependent variable: Depressive symptoms at follow-up. Model 1: Controlling for age and sex. Model 2: Model 1+ controlling for education, income, alcohol intake, smoking status, physical activity, and anemia.

**Table 4:** Odds ratio and 95% CI from multinomial logistic regression of having dietary exclusions at follow-up associated to depressive symptoms at baseline (binary measure) (N = 25,356).

Exclusions	No Dietary Exclusionsat Follow-Up	One Exclusion at Follow-Up	Two Exclusions at Follow-Up	Three Exclusions at Follow-Up	Four and More at Follow-Up
**Model 1**	Ref	1.62 (1.45–1.82)	1.79 (1.42–2.25)	2.34 (1.55–3.52)	5.56 (3.18–9.74)
**Model 2**	Ref	1.37 (1.22–1.54)	1.42 (1.12–1.79)	1.68 (1.10–2.57)	3.45 (1.93–6.16)

Dependent variable: Dietary exclusions at follow-up. Model 1: Controlling for age and sex. Model 2: Model 1+ controlling for education, income, alcohol intake, smoking status, physical activity, and anemia.

## Data Availability

The data that supports the findings of this study are available from the corresponding author upon reasonable request.

## Supplementary Materials

The following are available online at https://www.mdpi.com/2072-6643/12/9/2700/s1, Figure S1: Flowchart for the selection of participants, Table S1: OR and 95% CI from logistic regression for the odds of having depressive symptoms at follow-up (binary measure) associated to dietary exclusions at baseline in models 1 and 2 when the population is not restricted to those having complete data in model 3. Table S2a: Estimates and standard errors from general linear models for the associations of dietary exclusions at baseline with depressive symptoms at follow-up (continuous variable). Table S2b: Estimates and standard errors from general linear models for the associations of dietary exclusions at baseline with depressive symptoms at follow-up (continuous variable) but when the population is not restricted to those having complete data in model 3. Table S3: Odds and 95% CI from multinomial logistic regression of having dietary exclusions at follow-up associated to depressive symptoms at baseline (binary measure) in models 1 and 2 when the population is not restricted to those having complete data in model 3. Table S4a: Odds and 95% CI from multinomial logistic regression of having dietary exclusions at follow-up associated to depressive symptoms at baseline (continuous measure). Table S4b: Odds and 95% CI from multinomial logistic regression of having depressive symptoms at baseline with the association of dietary exclusions at follow-up (depressive symptoms analysed as a continuous variable) when the population is not restricted to those having complete data in model 3.

## References

[1] Sánchez-Villegas A., Delgado-Rodríguez M., Alonso A., Schlatter J., Lahortiga F., Majem L.S., Martínez-González M.A. (2009). Association of the Mediterranean dietary pattern with the incidence of depression: The Seguimiento Universidad de Navarra/University of Navarra follow-up (SUN) cohort. Arch. Gen. Psychiatry.

[2] Kim T.-H., Choi J.-Y., Lee H.-H., Park Y. (2015). Associations between dietary pattern and depression in Korean adolescent girls. J. Pediatr. Adolesc. Gynecol..

[3] Lassale C., Batty G.D., Baghdadli A., Jacka F., Sánchez-Villegas A., Kivimäki M., Akbaraly T. (2019). Healthy dietary indices and risk of depressive outcomes: A systematic review and meta-analysis of observational studies. Mol. Psychiatry.

[4] Lai J.S., Hiles S., Bisquera A., Hure A.J., McEvoy M., Attia J. (2014). A systematic review and meta-analysis of dietary patterns and depression in community-dwelling adults. Am. J. Clin. Nutr..

[5] Gougeon L., Payette H., Morais J., Gaudreau P., Shatenstein B., Gray-Donald K. (2015). Dietary patterns and incidence of depression in a cohort of community-dwelling older Canadians. J. Nutr. Health Aging.

[6] Vermeulen E., Knüppel A., Shipley M.J., Brouwer I.A., Visser M., Akbaraly T., Brunner E.J., Nicolaou M. (2018). High-sugar, high-saturated-fat dietary patterns are not associated with depressive symptoms in middle-aged adults in a prospective study. J. Nutr..

[7] Li Y., Lv M.-R., Wei Y.-J., Sun L., Zhang J.-X., Zhang H.-G., Li B. (2017). Dietary patterns and depression risk: A meta-analysis. Psychiatry Res..

[8] Matta J., Czernichow S., Kesse-Guyot E., Hoertel N., Limosin F., Goldberg M., Zins M., Lemogne C. (2018). Depressive symptoms and vegetarian diets: Results from the constances cohort. Nutrients.

[9] Michalak J., Zhang X.C., Jacobi F. (2012). Vegetarian diet and mental disorders: Results from a representative community survey. Int. J. Behav. Nutr. Phys. Act..

[10] Smith K.J., Sanderson K., McNaughton S.A., Gall S.L., Dwyer T., Venn A.J. (2014). Longitudinal associations between fish consumption and depression in young adults. Am. J. Epidemiol..

[11] Kingsbury M., Dupuis G., Jacka F., Roy-Gagnon M.-H., McMartin S.E., Colman I. (2016). Associations between fruit and vegetable consumption and depressive symptoms: Evidence from a national Canadian longitudinal survey. J. Epidemiol. Community Health.

[12] Elstgeest L.E., Visser M., Penninx B.W., Colpo M., Bandinelli S., Brouwer I.A. (2019). Bidirectional associations between food groups and depressive symptoms: Longitudinal findings from the Invecchiare in Chianti (InCHIANTI) study. Br. J. Nutr..

[13] Lavallee K., Zhang X.C., Michalak J., Schneider S., Margraf J. (2019). Vegetarian diet and mental health: Cross-sectional and longitudinal analyses in culturally diverse samples. J. Affect. Disord..

[14] Puccio F., Fuller-Tyszkiewicz M., Ong D., Krug I. (2016). A systematic review and meta-analysis on the longitudinal relationship between eating pathology and depression. Int. J. Eat. Disord..

[15] Voderholzer U., Hessler-Kaufmann J.B., Lustig L., Läge D. (2019). Comparing severity and qualitative facets of depression between eating disorders and depressive disorders: Analysis of routine data. J. Affect. Disord..

[16] Vulser H., Wiernik E., Hoertel N., Thomas F., Pannier B., Czernichow S., Hanon O., Simon T., Simon J.M., Danchin N. (2016). Association between depression and anemia in otherwise healthy adults. Acta Psychiatr. Scand..

[17] Goetz L.G., Valeggia C. (2017). The ecology of anemia: Anemia prevalence and correlated factors in adult indigenous women in Argentina. Am. J. Hum. Biol..

[18] Vulser H., Lemogne C., Boutouyrie P., Côté F., Perier M.-C., Van Sloten T., Hoertel N., Danchin N., Limosin F., Jouven X. (2020). Depression, antidepressants and low hemoglobin level in the Paris Prospective Study III: A cross-sectional analysis. Prev. Med..

[19] Zins M., Goldberg M. (2015). The French CONSTANCES population-based cohort: Design, inclusion and follow-up. Eur. J. Epidemiol..

[20] Morin A.J., Moullec G., Maiano C., Layet L., Just J.L., Ninot G. (2011). Psychometric properties of the Center for Epidemiologic Studies Depression Scale (CES-D) in French clinical and nonclinical adults. Rev. D’épidémiologie St. Publique.

[21] Hercberg S., Chat-Yung S., Chauliac M. (2008). The French national nutrition and health program: 2001–2006–2010. Int. J. Public Health.

[22] Plessz M., Kesse-Guyot E., Zins M., Matta J., Czernichow S. (2019). Poverty does not modify the association between perceived diet healthiness and adherence to nutritional guidelines in the Constances cohort (France). Appetite.

[23] Stubbs B., Koyanagi A., Schuch F.B., Firth J., Rosenbaum S., Veronese N., Solmi M., Mugisha J., Vancampfort D. (2016). Physical activity and depression: A large cross-sectional, population-based study across 36 low-and middle-income countries. Acta Psychiatr. Scand..

[24] Wagner A., Simon C., Evans A., Ferrières J., Montaye M.l., Ducimetière P., Arveiler D. (2002). Physical activity and coronary event incidence in Northern Ireland and France: The Prospective Epidemiological Study of Myocardial Infarction (PRIME). Circulation.

[25] Pino E.C., Damus K., Jack B., Henderson D., Milanovic S., Kalesan B. (2018). Adolescent socioeconomic status and depressive symptoms in later life: Evidence from structural equation models. J. Affect. Disord..

[26] Stage F.K., Carter H.C., Nora A. (2004). Path analysis: An introduction and analysis of a decade of research. J. Educ. Res..

[27] Blanco C., Hoertel N., Wall M.M., Franco S., Peyre H., Neria Y., Helpman L., Limosin F. (2018). Toward Understanding Sex Differences in the Prevalence of Posttraumatic Stress Disorder: Results From the National Epidemiologic Survey on Alcohol and Related Conditions. J. Clin. Psychiatry.

[28] Wolfe A.R., Ogbonna E.M., Lim S., Li Y., Zhang J. (2009). Dietary linoleic and oleic fatty acids in relation to severe depressed mood: 10 years follow-up of a national cohort. Prog. NeuroPsychopharmacol. Biol. Psychiatry.

[29] Wang J., Um P., Dickerman B.A., Liu J. (2018). Zinc, magnesium, selenium and depression: A review of the evidence, potential mechanisms and implications. Nutrients.

[30] Molendijk M., Molero P., Sánchez-Pedreño F.O., Van der Does W., Martínez-González M.A. (2018). Diet quality and depression risk: A systematic review and dose-response meta-analysis of prospective studies. J. Affect. Disord..

[31] Appleton K., Woodside J., Yarnell J., Arveiler D., Haas B., Amouyel P., Montaye M., Ferrieres J., Ruidavets J., Ducimetiere P. (2007). Depressed mood and dietary fish intake: Direct relationship or indirect relationship as a result of diet and lifestyle?. J. Affect. Disord..

[32] Collin C., Assmann K., Deschasaux M., Andreeva V., Lemogne C., Charnaux N., Sutton A., Hercberg S., Galan P., Touvier M. (2017). Statut en vitamine D et symptômes dépressifs récurrents dans la cohorte française SU. VI. MAX. Nutr. Clin. Métabolisme.

[33] Andreeva V.A., Galan P., Torres M., Julia C., Hercberg S., Kesse-Guyot E. (2012). Supplementation with B vitamins or n–3 fatty acids and depressive symptoms in cardiovascular disease survivors: Ancillary findings from the SUpplementation with FOLate, vitamins B-6 and B-12 and/or OMega-3 fatty acids (SU. FOL. OM3) randomized trial. Am. J. Clin. Nutr..

